# A Systematic Evaluation of Integration Free Reprogramming Methods for Deriving Clinically Relevant Patient Specific Induced Pluripotent Stem (iPS) Cells

**DOI:** 10.1371/journal.pone.0081622

**Published:** 2013-11-26

**Authors:** Pollyanna A. Goh, Sara Caxaria, Catharina Casper, Cecilia Rosales, Thomas T. Warner, Pete J. Coffey, Amit C. Nathwani

**Affiliations:** 1 Research Department of Haematology, University College London Cancer Institute, University College London, London, United Kingdom; 2 National Health Service Blood and Transplant Unit, National Health Service, London, United Kingdom; 3 Reta Lila Weston Institute of Neurological Studies, Institute of Neurology, University College London, London, United Kingdom; 4 Ocular Biology and Therapeutics, Institute of Ophthalmology, University College London, London, United Kingdom; 5 Katharine Dormandy Haemophilia Centre and Thrombosis Unit, Royal Free Hospital, London, United Kingdom; Southern Illinois University School of Medicine, United States of America

## Abstract

A systematic evaluation of three different methods for generating induced pluripotent stem (iPS) cells was performed using the same set of parental cells in our quest to develop a feeder independent and xeno-free method for somatic cell reprogramming that could be transferred into a GMP environment. When using the BJ fibroblast cell line, the highest reprogramming efficiency (1.89% of starting cells) was observed with the mRNA based method which was almost 20 fold higher than that observed with the retrovirus (0.2%) and episomal plasmid (0.10%) methods. Standard characterisation tests did not reveal any differences in an array of pluripotency markers between the iPS lines derived using the various methods. However, when the same methods were used to reprogram three different primary fibroblasts lines, two derived from patients with rapid onset parkinsonism dystonia and one from an elderly healthy volunteer, we consistently observed higher reprogramming efficiencies with the episomal plasmid method, which was 4 fold higher when compared to the retroviral method and over 50 fold higher than the mRNA method. Additionally, with the plasmid reprogramming protocol, recombinant vitronectin and synthemax® could be used together with commercially available, fully defined, xeno-free essential 8 medium without significantly impacting the reprogramming efficiency. To demonstrate the robustness of this protocol, we reprogrammed a further 2 primary patient cell lines, one with retinosa pigmentosa and the other with Parkinsons disease. We believe that we have optimised a simple and reproducible method which could be used as a starting point for developing GMP protocols, a prerequisite for generating clinically relevant patient specific iPS cells.

## Introduction

Mature somatic cells can be reprogrammed to a pluripotent state through ectopic expression of key transcription factors, in a process known as induced pluripotency. The resulting induced pluripotent stem (iPS) cells have unlimited proliferative potential while maintaining the capacity to differentiate into any cell type. These pluripotent characteristics coupled with the ability to derive iPS cells from adult patient cells, have made iPS cells a valuable tool for the *in vitro* modelling of many human diseases, drug discovery and may potentially serve as an unlimited source of cells for regenerative medicine. While many patient specific iPS cell lines have already been derived, most have been generated using genome integrating methods which raises concerns of insertional mutagenesis and continued expression of potentially oncogenic proteins by the integrated transgenes [1]. These concerns are particularly important when considering clinical translation. It is therefore desirable to generate iPS cells using protocols that dispense with the need for integrating viral vectors, whilst being robust and fully compliant with good manufacturing practice (GMP) requirements. Several integration-free methods have been reported, including episomal plasmids [2], recombinant proteins [3], temperature sensitive sendai virus [4], synthetic mRNA [5] and miRNA [6] approaches, each with unique advantages and disadvantages and reprogramming efficiencies. The initial report using OriP/EBNA-1 based episomal plasmids showed that it is a technically simple method of reprogramming though, extremely inefficient (1-3 colonies from 10^6^ input cells) [2]. However, subsequent reports have shown that the replacement of SV40 large T antigen, Nanog and c-Myc, with a shp53 and L-Myc can improve reprogramming efficiencies over 10 fold [1]. These studies demonstrate that reprogramming using episomal plasmids is a viable approach for generating integration free iPS cells. Another interesting method of reprogramming showed the use of a synthetic mRNA cocktail including Oct4, Sox2, Klf4, c-Myc and Lin28 to yield reprogramming efficiencies of up to 4.4%, the highest reported thus far [5]. The RNA based method is not associated with chromosomal integration, which is an important safety attribute.

However, a systematic evaluation of the reprogramming methods is yet to be conducted using the same set of cell lines and under the same culture conditions. Another major hurdle for the clinical translation of iPS cells is the need to use fully defined, xeno-free reagents. Despite progress in developing protocols using xeno-free reagents, these methods are still reliant on the use of human feeders and viral vectors [7-10]. Here, our aim was to compare the reprogramming efficiencies of the retrovirus, episomal plasmid and mRNA reprogramming methods using the same set of transformed and primary patient cell lines to determine which method is most suitable for clinical translation. Our data shows that the non-viral, episomal plasmid method is most efficient and robust at reprogramming primary human fibroblasts even under feeder free conditions and therefore, would be most suitable for the production of GMP grade iPS cells.

## Materials and Methods

### Ethics statement

All human primary fibroblast cells were generated *in vitro* after written informed consent using protocols approved by the Royal Free research ethics committee, Royal Free Hospital, London, UK. All animal work was performed under the authority of the UK Home Office Project and Personal Licenses regulations and was compliant with the guidelines of the University College London Ethics Committee.

### Isolation of dermal fibroblasts and Cell culture

All reagents were sourced from Life Technologies UK, unless otherwise stated. All cells were cultured in a humidified incubator at 37°C in 5%CO_2_. Consent was obtained from patient RDP1, 49 years old patient with rapid onset parkinsonism dystonia (RDP); patient RDP2, 31 years old with RDP; patient PD1, 70 years old with Parkinsons disease; patient RP2 with retinosa pigmentosa and; control CTL1, an 81 year old healthy volunteer for tissue collection. An approximately 3-6mm skin punch biopsy was collected by a trained physician under local anaesthetic and fibroblasts generated following previously published protocols [11]. BJ fibroblast cell line (Stemgent) and established fibroblast cell lines were cultured in fibroblast medium consisting of high glucose DMEM (PAA cell laboratories) with 10% foetal bovine serum, 1mM non-essential amino acids (NEAA) and penicillin-streptomycin (PAA cell laboratories). When cells reached 80% confluency, fibroblasts were detached using TrypLE express. Splits of 1:3-1:5 were performed every 4-5 days with medium being refreshed every 2-3 days. For xeno-free culture of established fibroblast cell lines, fibroblast medium was replaced with TheraPeak MSCGM-CD medium (Lonza). H1 hES cell line (gift of Tariq Enver, UCL Cancer Institiute, London UK) [12] and established iPS cell lines were maintained in one of the following combinations: irradiated newborn human foreskin foetal fibroblasts feeders (Nuff, GlobalStem) with hUES medium (20% knock out serum replacement in DMEM/F12 with 1mM glutaMAX, 1mM NEAA, penicillin-streptomycin, 0.1mM β-mercaptoethanol and 10ng/ml β-Fgf) or; hES cell qualified matrigel (BD Biosciences) and mTESR1 (Stemcell Technologies) or; recombinant vitronectin and essential 8 (E8) medium. Medium was refreshed every day and cells were passaged 1:3-1:5 using collagenase type IV, dispase (Stemcell Technologies) or gentle cell dissociation reagent (Stemcell Technologies). Methods for passaging human pluripotent stem cells have been described elsewhere [13].

### Reprogramming fibroblasts

Shinya Yamanaka’s pMXs-based retroviral vectors were obtained from Addgene (Addgene numbers 17217, 17218, 17219, 17220) and have been previously described [14]. Platinum A amphotrophic packaging cell line (Cell biolabs) was used to generate retroviruses. 10^5^ target fibroblasts were seeded in a single well of a 6 well plate the day prior to transduction. The next day, viral supernatants containing equal volume of each factor was supplemented with 8µg/ml of polybrene (Sigma-Aldrich) and added to each well and left overnight. Fresh retroviral supernatants were added the following day for a second round of transduction. Four days after the initial transduction, cells were harvested using trypLE express and transferred to a 10cm dish seeded with 10^6^ Nuff feeders. The next day, medium was changed to hUES cell medium.

Two different sets of episomal plasmids obtained from Addgene were used in experiments. James Thomson episomal plasmids: pEP4-EO2S-ET2K (SET2K, Addgene number 20927) and pEP4-EO2S-CK2M-EN2L (EN2L, Addgene number 20924) and Shinya Yamanaka episomal plasmids: pCXLE-hOct3/4-shp53-F (Addgene number 27077), pCXLE-hSK (Addgene number 27078), pCXLE-hUL (Addgene number 27080) and pCXLE-EGFP (Addgene number 27082) have been previously described [1,2]. Early passage fibroblasts (less than 12 passages) in growth phase were used for all experiments. Fibroblasts were harvested with TrypLE express and the cell pellet washed once in PBS. 10^6^ cells were counted and re-suspended in nucleofector solution supplied in the Amaxa Nucleofector kit R (Lonza). Episomal plasmids were added to the cell suspensions at various concentrations, Thomson plasmids: SET2K 3.2µg and EN2L 7.2µg per reaction and Yamanaka plasmids at 1µg each per reaction. Cell suspensions were transfected using program U-023 on a Nucleofector (I) 2b device. Immediately following transfection, cells were re-suspended in fibroblast medium and transferred to a 10cm tissue culture dish coated with 0.1% gelatine (Sigma-Aldrich). Daily medium changes using fresh fibroblast medium supplemented with 0.5mM sodium butyrate (Sigma-Aldrich) was begun the following day. At day 7 post transfection, cells were counted using trypan blue and 10^5^ viable cells transferred to a 10cm dish seeded with 10^6^ Nuff feeders. Alternatively for feeder free derivation, 2×10^5^ viable cells were seeded into one well of a 6 well plate coated with matrigel (BD Biosciences), recombinant vitronectin, laminin-521 (Biolamina) or Synthemax-R (Corning). The next day, fibroblast medium was changed to hUES cell medium, mTESR1 or Essential 8 (E8) medium (E8 from Life technologies or TeSR-E8 from Stemcell technologies) supplemented with 0.5mM sodium butyrate. By day 12 post transfection, sodium butyrate treatment was stopped and conditioned hUES medium/mTESR1 or E8 used instead.

The mRNA reprogramming kit and Pluriton reprogramming medium (Stemgent) were used as per manufacturer’s instructions for both feeder dependent (Nuffs) and feeder free (matrigel) derivation. 

At day 30 post transduction/transfection, cells were either fixed for whole plate alkaline phosphatase staining or live stained with TRA-1-81 to pick iPS colonies. TRA-1-81 primary antibody (Santa Cruz Technologies) was diluted 1:100 and goat anti mouse IgG/IgM conjugated Alexa488 secondary antibody diluted 1:400 in pre-warmed DMEM/F12. After 30 mins, plates were washed 2 times with DMEM/F12 and fresh hUES/mTESR1/E8 medium added during imaging. The number of TRA-1-80 positive colonies were counted and several colonies were picked and transferred to organ well culture dishes (BD biosciences) or 12 well plates coated with matrigel or seeded with 10^4^ Nuffs. After 4-5 mechanical passages, iPS cells were switched to enzymatic passaging. Feeder dependent iPS cell lines were transitioned and maintained in matrigel and mTESR1 medium from passages 8-9.

### Characterisation of iPS cells

Immunophenotyping entailed washing the cells once with PBS and then fixed with 4% paraformadehyde (Electron microscopy services) for 20 mins. Cells were washed 3 times with PBS and then stored at 4°C in PBS. Prior to immunostaining, cells were blocked for 30 mins with 5% goat serum, 0.1% bovine serum albumin (Sigma-Aldrich) in PBS. If performing nuclear staining, cells were permeabilised with ice-cold 100% methanol for 5 mins prior to blocking. Primary antibodies Oct3/4, TRA-1-60, SSEA-1, SSEA-3 and SSEA-4 (Santa Cruz Biotechnologies) were used at a 1:100 dilution and used to stain cells at room temperature for one hour. Primary antibodies were washed off 3 times with PBS and then goat anti-mouse IgG/IgM Alexa 488 diluted 1:400 added. Secondary antibody was incubated at room temperature for 30 mins. Cells were washed 3 times with PBS and then DAPI (Sigma-Aldrich) added for 5-10 mins. Cells were washed 3 times with PBS and immediately visualised. For alkaline phosphatase staining, cells were stained with the alkaline phosphatase staining kit (Millipore) as per manufacturer’s instructions.

To demonstrate spontaneous *in vitro* differentiation, iPS cells were grown to confluency and harvested by dispase. Cells were re-suspended in hES medium without β-Fgf and transferred to non-tissue culture treated 6 well plates (Grenier Bio One). Medium was changed every 3-4 days. Day 12 embryoid bodies (EBs) were transferred to a 12 well plate coated with 0.1% gelatine and cultured for a further 12 days in fibroblast medium. Differentiated cells were harvested with TRIzol and stored at -80°C until phenol chloroform RNA extraction. 1µg of RNA was used for subsequent reverse transcriptase reactions with Superscript III first strand synthesis system. 1µl of cDNA was used per PCR reaction with GoTaq green mastermix (Promega). Primers and conditions for RT-PCR have been previously described [15].

Pluripotency and trilineage differentiation potential was also assessed using the TaqMan® hPSC Scorecard^TM^ kit 384w following manufacturer’s instructions and run on a ViiA 7 system. Data analysis was performed using the cloud based TaqMan® hPSC Scorecard^TM^ analysis software.

Genomic DNA analysis entailed the use of the DNeasy blood and tissue kit (Qiagen) as per manufacturer’s instructions. 100-200ng of DNA was used per PCR reaction with GoTaq green mastermix. Primers and conditions for PCR are listed in table 1 or have been previously described [2]. For STR analyses, PCR products were run on a 3.5% agarose gel at 30V/cm. G-banding analysis on iPS cell colonies was performed by either the Haematology Cellular & Molecular Diagnostic Service, Great Ormond Street Hospital & UCL Institute of Child Health or TDL genetics, The Doctors laboratory, London.

**Table 1:** The plasmid reprogramming efficiencies of fibroblasts cultured in xeno-free, feeder-free conditions (E8 and vitronectin).

**Cell name**	**GFP transfection efficiency**	**Number of TRA-1-81+ colonies^*1*^**	**Reprogramming efficiency^*2*^**
BJ control	44.02%	187±16.97	0.21%
RPD1	39.10%	113.5±13.44	0.15%
RPD2	42.52%	159.5±3.54	0.19%
PD1	45.03%	206±4.0	0.23%
RP2	42.20%	217.5±4.5	0.26%

*1* n=2, mean±SE

*2* reprogramming efficiency% = number of iPS colonies/number of transduced or transfected cells.

For *in vivo* pluripotency, iPS cells were grown to confluency and harvested by dispase. Cell pellets were re-suspended in 30% matrigel and 70% mTESR1. One confluent 6 well plate was used per animal. 6-8 week old NOD-SCID mice were anaesthetised by isoflurane inhalation and 50µl of the iPS cell suspension was injected into each testis capsule. Analgesia (Carprofen, 5mg/kg) was administered intraperitonally following surgery to minimise pain. After 10-12 weeks, animals were sacrificed by carbon dioxide asphyxiation and teratomas excised and fixed in Histochoice (Amresco). Histological processing and H&E staining were performed by either, The Research Department of Pathology, Faculty of Medical Sciences, University College London or The Research Department of Oncology, UCL Cancer Institute.

## Results

### Comparison of reprogramming methods using the BJ fibroblast cell line

**Figure 1:**
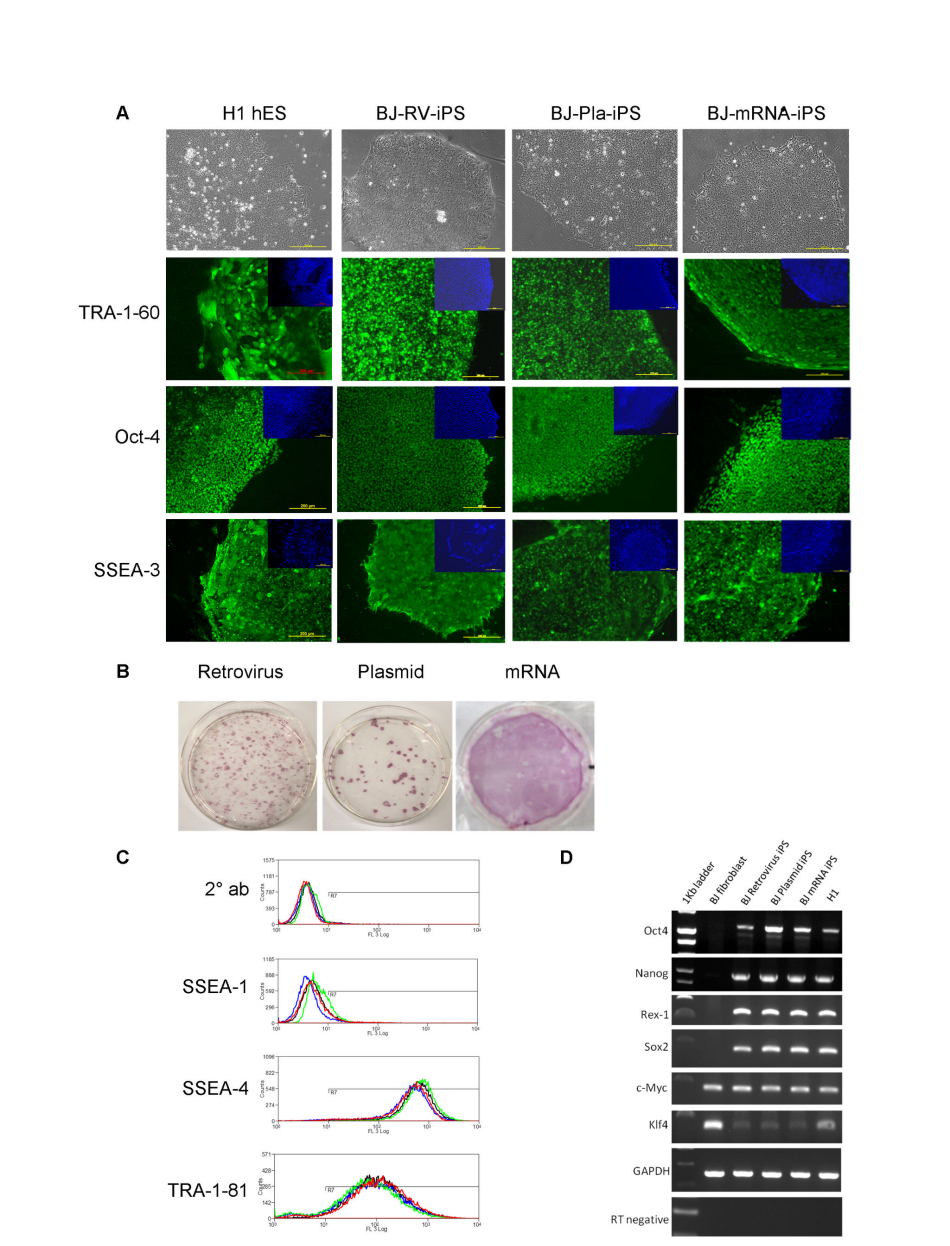
Characterisation of established iPS lines derived from BJ fibroblasts using retrovirus, plasmids and mRNA. A) Representative phase images of established iPS and H1 cell lines. Immunostaining with pluripotency markers (green) and counter staining with DAPI (blue). Scale bar is 200µm. B) Alkaline phosphatase staining of whole plates/wells at days 30 (retrovirus and plasmid) and day 20 (mRNA). C) Flow cytometry analysis with pluripotency markers SSEA-4 and TRA-1-81. SSEA-1 is a negative marker of human pluripotent stem cells. Green line denotes H1, red line denotes BJ-RV-iPS, green line denotes BJ-Pla-iPS and black line denotes BJ-mRNA-iPS. D) RT-PCR analysis for expression of key pluripotency genes.

The standard Yamanaka retrovirus protocol was first used to determine the baseline reprogramming efficiency of BJ fibroblasts that could be achieved in our laboratory. Transduction with the 4 factor retroviruses gave 112 TRA-1-81 positive iPS colonies by day 30. In parallel experiments, the number of transduced cells was estimated to be 85.90%, as assessed by a GFP encoding retroviral vector. (Figure S1A). Therefore, the estimated reprogramming efficiency with retroviral vectors was 0.2% (Figure 1A). Several retrovirus iPS cell lines (BJ-RV-iPS) were established and one line used as a standard for further characterisation.

Next, we compared the Thomson episomal plasmids (encoding Oct4, Sox2, Klf4, c-Myc, Nanog, Lin28 and SV40 large T antigen) with the Yamanaka episomal plasmids (encoding Oct4, Sox2, Lin28, L-Myc and p53 shRNA) following their published protocols. Irradiated human neonatal foreskin fibroblasts (Nuffs) were used as a feeder layer for both plasmid combinations in our initial studies. The transfection efficiency of BJ fibroblasts using the pCXLE GFP plasmid construct was approximately 36±16% (n=4) at 24 hours after electroporation (Figure S1B). Morphology changes in fibroblasts were observed in induced cells with the Thomson plasmids as early as 7 days post transfection, while for the Yamanaka plasmids, changes were first observed at days 10-12. However, by days 25-30 more iPS-like colonies emerged from Yamanaka plasmids. *Bona fide* iPS colonies were identified by TRA-1-81 live staining. An average of 43±29 colonies were observed for Yamanaka plasmids compared with only 10.5±2.5 positive colonies for Thomson plasmids (n=2). After taking into consideration the transfection efficiency, the computed reprogramming efficiency of the Yamanaka plasmid combination was found to be over 10 fold greater (0.012±0.008% Yamanaka vs 0.003±0.001% Thomson) at generating iPS cells consistent with previous reports [1,16]. The Yamanaka method was, therefore, used in all subsequent experiments. Since feeder cells can vary substantially in quality, we sought to eliminate their use in our experiments. We replaced the human feeders with matrigel and observed 76.33±5.6 TRA-1-81 positive colonies (n=3), which resulted in a higher reprogramming efficiency than the feeder dependant protocol (0.103±0.008% Yamanaka feeder free vs 0.012±0.008% Yamanaka feeder dependent). Several iPS lines were established and one line used for further characterisation (BJ-pla-iPS).

With the mRNA based reprogramming method, the transfection efficiency of BJ fibroblasts with the mRNA cocktail was highly impressive as it approached 100% as assessed using the Axiovision software, (5 images/transfected tissue culture wells compared with hoescht H33342 staining) (Figure S1C). We observed early (after 3 days) morphology changes of BJ fibroblasts from a spindle-like to a cobble stone-like appearance and iPS cell like colonies began to emerge after 12 days of transfections, which is significantly earlier than observed with the plasmid and retroviral reprogramming methods. Live TRA-1-81 antibody staining of colonies at day 18, revealed 189 positive colonies, equating to a reprogramming efficiency of 1.89%. However, when we replaced feeders with matrigel, the reprogramming efficiency with the mRNA protocol decreased to 0.22%. Several mRNA derived iPS lines were established and one line was used for further characterisation (BJ-mRNA-iPS).

**Figure 2:**
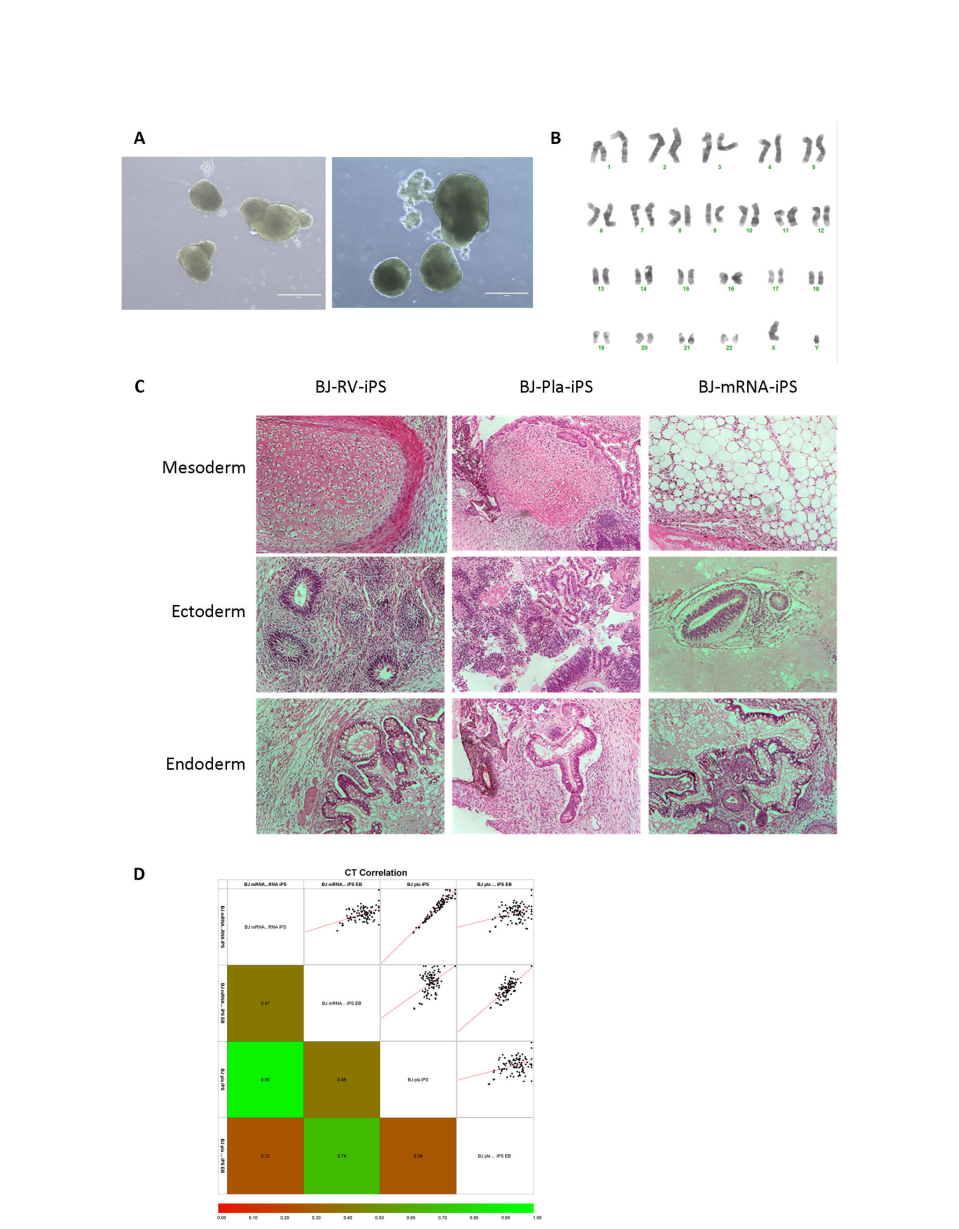
Pluripotency of established iPS lines and detecting the genomic integration of episomal plasmids. A) Representative images of embryoid bodies (EBs) generated from BJ-pla-iPS (left) and BJ-mRNA-iPS (right). B) Repsentative image of karyotype 46, XY. BJ-pla-iPS cells at passage 15 is shown. C) H&E stained slides of teratomas formed from injections of iPS cells into the testis capsule of NOD-SCID mice. Tissue derivatives indicative of the three germ lineages were observed. D) Human pluripotent stem cell scorecard assay results comparing BJ-pla-iPS and BJ-mRNA-iPS lines.

The iPS lines established from BJ fibroblasts using retrovirus, episomal plasmids and mRNA all showed typical hES-like morphology with large nucleus to cytoplasm ratio, highly visible nucleoli and growth as compact colonies (Figure 1A). Immuno-staining analyses indicated that all iPS lines expressed typical hES cell antigens including alkaline phosphatase, SSEA-3, TRA-1-60 and Oct4 (Figure 1A, 1B). Flow cytometric analysis further demonstrated that 80-90% of the populations expressed cell surface antigens, SSEA-4 and TRA-1-81 but did not express SSEA-1, a mouse marker of pluripotency, which is comparable to the hES cell profile (Figure 1C). RT-PCR analysis showed the expression of endogenous pluripotency genes including Oct4, Sox2, Nanog and Rex1 in all iPS lines in contrast with the parental BJ fibroblasts (Figure 1D). All iPS lines could be used to generate embryoid bodies (EBs) and at day 12, EBs were transferred to gelatine coated dishes for further spontaneous differentiation (Figure 2A). Quantitative RT-PCR revealed the down regulation of pluripotency genes, Oct4 and Nanog in differentiated cells while lineage specific genes Pax6, Mixl1 and Cdx2 were up-regulated, demonstrating the *in vitro* pluripotency of these cells (Figure S2A,B). When iPS cell lines were injected into the testis capsule of immune compromised mice, teratomas formed which consisted of tissue derivatives indicative of the three germ lineages confirming the *in vivo* pluripotency of the iPS cell lines derived (Figure 2C). Furthermore, cytogenetic analysis revealed normal karyotypes (Figure 2B), while DNA fingerprinting confirmed the parental origin of iPS lines as being derived from BJ fibroblasts (Figure S2C). Recently, Meissner’s group reported the generation of a scorecard to predict the pluripotency and germ layer bias of human pluripotent stem cells [17]. We used this scorecard to compare the BJ iPS lines generated using episomal plasmids and mRNA. Scores of 0.39 and 0.98 (scores between -0.5 and 1 are deemed the same) were obtained for the undifferentiated iPS cells respectively, which indicate that both lines expressed pluripotency markers at levels comparable to the reference standard. When testing the trilineage potential of these 2 iPS lines, day 7 embryoid bodies were generated, analysed and found to be good general purpose iPS cell lines without lineage bias (Figure 2D). These scorecard results validate our results from our *in vitro* differentiation and teratoma assays.

### Comparison of reprogramming methods using primary patient cell lines

Primary fibroblasts derived from two patients (RDP1 and RDP2) and a healthy volunteer (CTL1) were derived from skin biopsy samples obtained following informed consent. All 3 lines could be readily transduced with retroviral vectors, with GFP efficiencies of > 90% (Figure S1A). The number of TRA-1-81 positive iPS colonies observed with the retroviral method for RDP1 was 49, RDP2 was 31 and CTL1 was 12. Taking into consideration the GFP transduction efficiencies, the computed reprogramming efficiencies were 0.06%, 0.04% and 0.02% respectively. Using the Yamanaka episomal plasmid method under feeder free conditions, patient iPS lines were generated at efficiencies equal to or greater than that seen when reprogramming with retrovirus. RDP1 (0.09±0.02%) and RDP2 (0.16±0.06%) were more efficiently reprogrammed than CTL1 (0.02±0.0003%). These differences in reprogramming efficiency did not appear to be related to disease status but suggested a correlation with the age of the patient as reported before [18,19]. In contrast, attempts to reprogram RDP1 and RDP2 cell lines on 5 separate occasions with the mRNA method was largely unsuccessful even though parallel control studies with the BJ fibroblast cell line consistently showed a high reprogramming efficacy. Several variations of this protocol were attempted including different fibroblast seeding densities (ranging from 10^4^to 10^5^), hypoxia (5% O_2_) and the addition of a Stemgent proprietary microRNA cocktail. Three TRA-1-81 positive iPS-like colonies emerged from RDP2 cells after 25 days in culture using conditions in which the mRNA cocktail was combined with microRNA but not with the other conditions. Unfortunately, these 3 colonies could not be expanded into stable iPS lines as they began differentiating after the first passage.

**Figure 3:**
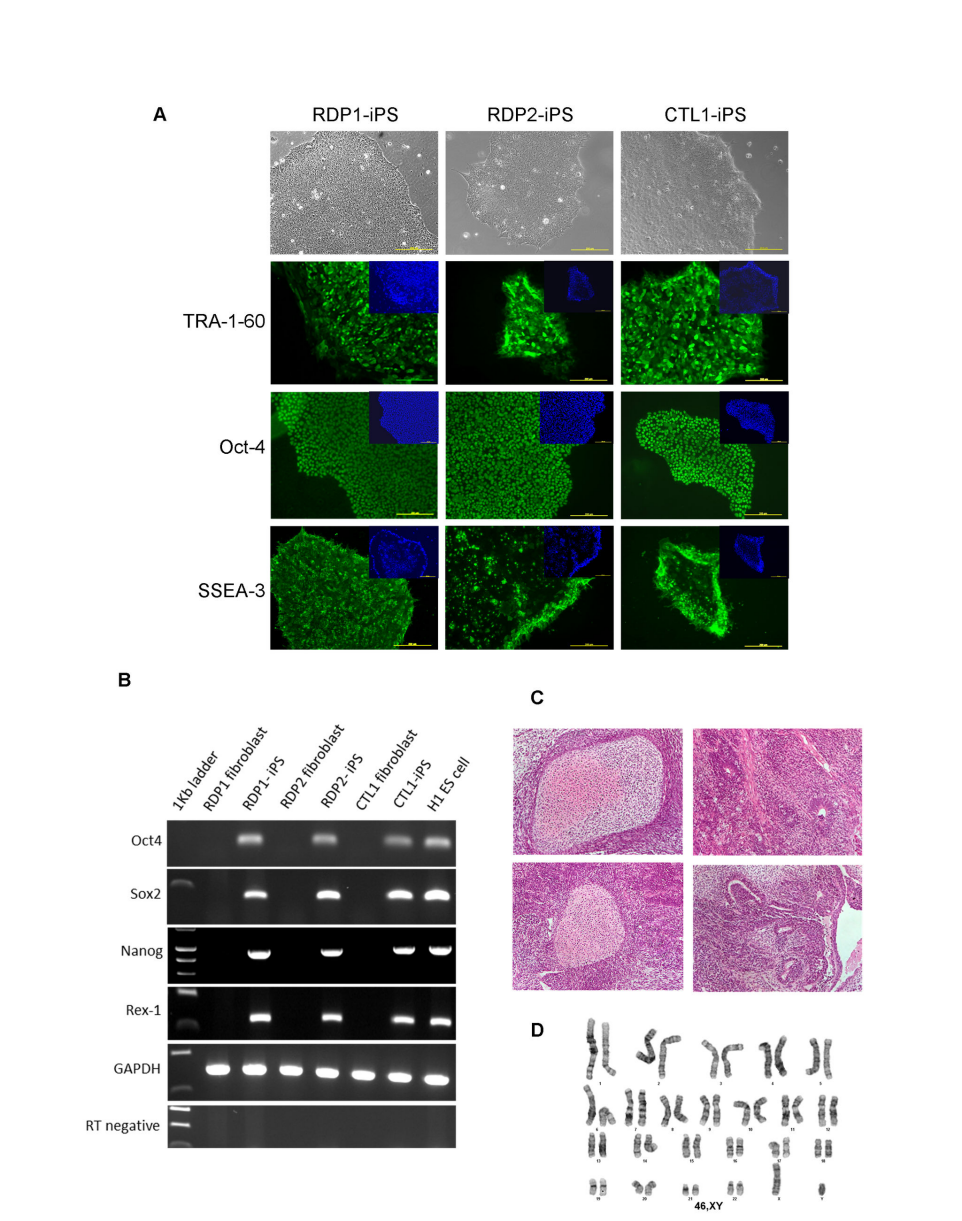
Characterisation of established patient specific iPS lines. A) Phase images of iPS lines established from patients RDP1, RDP2 and CTL1 using episomal plasmids. Immunostaining for markers of pluripotency shown in green. Cells were counter stained with DAPI (blue). Scale bar is 200µm. B) RT-PCR analysis showing expression of endogenous pluripotency genes: Oct4, Sox2, Nanog and Rex-1. C) Representative slides showing H&E staining of teratomas formed from injection of patient iPS lines into NOD SCID mice. Tissue derivatives indicative of the three germ lineages are shown. D). Representative karyotype, 46 XY of CTL1-iPS line analysed at passage 42.

**Figure 4:**
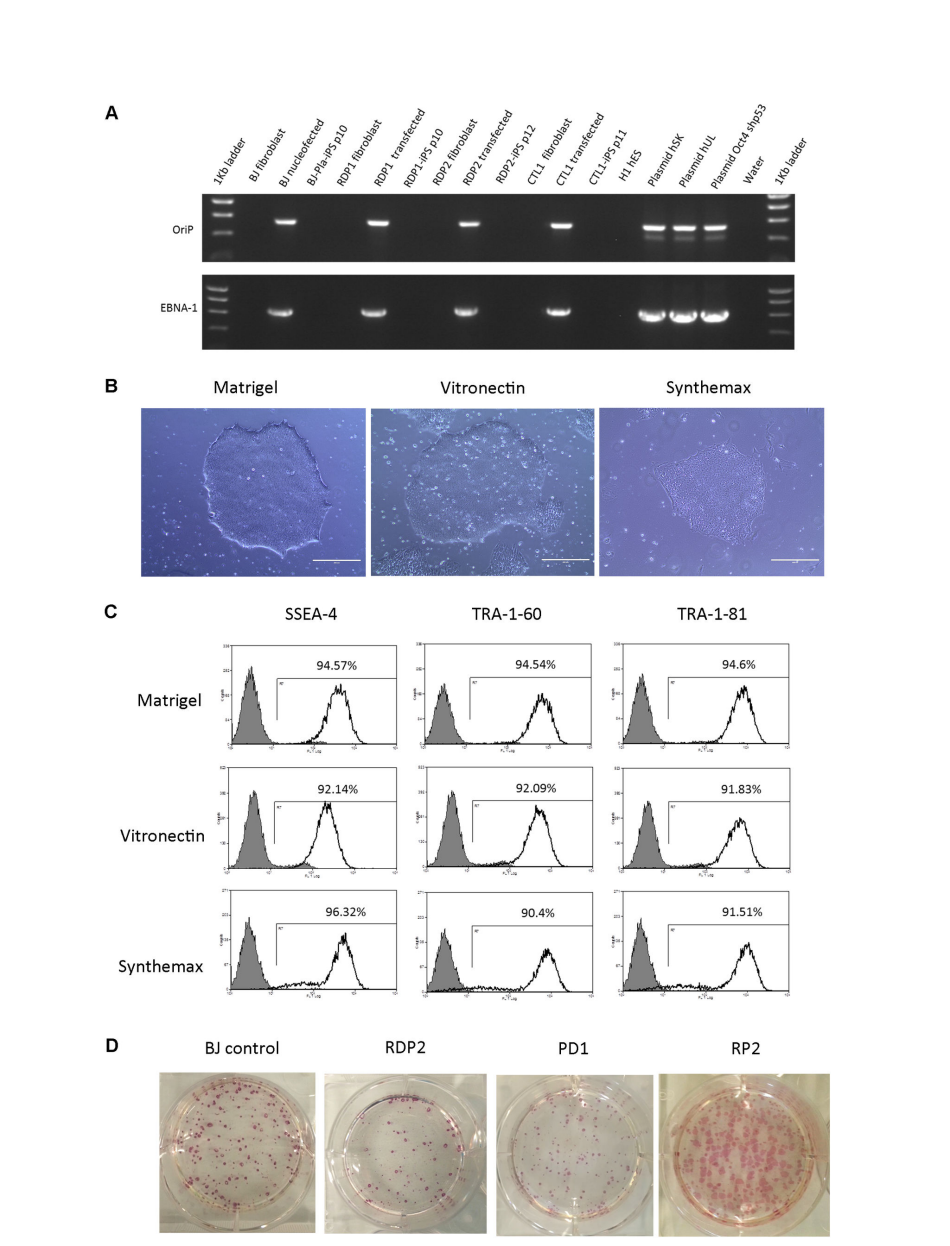
Comparison of different culture conditions during plasmid based reprogramming. A) Genomic integration of episomal plasmids. EBNA-1 and OriP sequences could not be detected in established iPS lines by passage 10. B) Morphology of RDP2-iPS cell lines derived in E8 medium, using different extracellular matrices. Scale bar is 400µm. C) Flow cytometric analysis of SSEA-4, TRA-1-81 and TRA-1-60 expression in RDP2-iPS cell lines derived on different matrices. Grey shaded areas denote the secondary antibody alone control. D) Whole well alkaline phosphatase staining of BJ, RDP2, PD1 and RP2 fibroblasts at day 30 of reprogramming using E8 medium and vitronectin.

The patient specific iPS lines derived using plasmids in feeder free conditions were characterized using the standard pluripotency assays. These patient iPS lines expressed key markers of pluripotency as determined by RT-PCR and immunostaining, and formed teratomas when injected into SCID mice (Figure 3A-C). All patient iPS lines had normal karyotypes, despite extensive passaging (Figure 3D). DNA fingerprinting confirmed their paternity (Figure S2C). Most importantly, genomic PCR analyses which can detect the EBNA-1 and OriP sequences with a sensitivity of 1 copy in 10^5^ cells showed that these plasmid sequences could not be found in iPS lines from passage 10 onwards (Figure 4A). This indicates that all the iPS lines generated using plasmids (BJ-pla-iPS, RDP1-iPS, RDP2-iPS and CTL1-iPS) were indeed integration-free.

### Using a xeno-free method for generating patient iPS cell lines

To bring iPS cells a step closer to the clinic, protocols for generating patient iPS cells ideally need to be feeder free, integration free and derived using fully defined xeno-free reagents. As the episomal plasmid method for iPS cell derivation was the most efficient and robust when reprogramming primary patient fibroblasts, we attempted to optimise this protocol further to achieve a method that could be amenable to GMP processes. We first replaced matrigel with laminin-521 but failed to generate any iPS cell colonies (data not shown). Next, we used recombinant vitronectin and Synthemax®, a novel vitronectin-mimicking surface and both allowed robust generation of RDP2 specific iPS colonies at efficiencies (0.18±0.03% and 0.27±0.06% respectively) comparable to matrigel (0.15±0.05%, n=3) [16-18]. To eliminate the use of mTESR1 medium which contains bovine serum albumin, we adopted the xeno-free essential 8 (E8) medium and recombinant vitronectin system developed by the Thomson lab [20] and observed an improved reprogramming efficiency of 0.23±0.08% (n=3) using RDP2 fibroblasts but this was not significant compared with mTESR1/matrigel system. To demonstrate the robustness of our feeder-free plasmid protocol using Thomson’s E8/vitronectin culture conditions, we further reprogrammed 3 more primary patient cell lines: RPD1, PD1 and RP2 all at high efficiencies (see Table 1 and Fig 4D). Several patient (RP2) iPS lines derived in these conditions were picked and continually expanded in E8 medium and either vitronectin, Synthemax® or matrigel matrices. Patient iPS cells expanded in vitronectin and Synthemax® appeared to be more flattened with less defined, rounded borders compared with iPS cells cultured in matrigel which matched previous observations (Fig 4B) [20]. To determine whether the morphology differences observed between vitronectin, Synthemax® and matrigel also led to differences in the expression of key markers of pluripotency, flow cytometry analysis was performed. The percentage of SSEA-4, TRA-1-60 and TRA-1-81 surface antigens expressed on iPS lines derived and maintained in matrigel was higher than with vitronectin and Synthemax®, but this was not statistically significant (Fig 4C). While the morphology of iPS colonies grown in vitronectin and synthemax® is distinct, our results show that these two matrices have the capacity to support both plasmid reprogramming and the growth of pluripotent stem cells which is consistent with previous findings [20]. In a step to further make our protocol more amenable to GMP processes, we eliminated FBS present in our fibroblast medium by using MSCGM-CD medium, a GMP qualified medium. Using MSCGM-CD medium combined with sodium butyrate during the first 7 days of the reprogramming process subsequently followed by culture on recombinant vitronectin and in E8 medium, we could also reprogram cells from patient RP2 at efficiencies equivalent to that observed when using foetal calf serum based medium (results not shown). 

## Discussion

Since Takahashi and Yamanaka’s landmark study, there have been an exponential number of publications describing the generation of iPS cells. While iPS cells can now be generated routinely in most stem cell laboratories, there is still no consensus on the best method to move forward with in a clinical setting. This question must be answered quickly so that we can turn our attention towards deriving the first GMP grade iPS cell lines. Currently, there are several limitations to the use of iPS cell derived progenitors in the clinic. Firstly, the use of genome integrating viruses needs to be eliminated to decrease the risk of oncogenesis. Secondly, the iPS cells must be generated using fully defined xeno-free reagents to comply with GMP requirements. In this study we performed a side-by-side comparison of the most clinically relevant reprogramming methods to identify one that is most suitable for the generation of iPS cells using GMP conditions. Our studies show that the Yamanaka plasmid based method is as efficient as the standard retroviral method at reprogramming an established cell line like BJ fibroblasts as well as primary fibroblasts derived from patients with a neurodegenerative condition or an elderly healthy volunteer. Importantly, the plasmid vectors were not detectable in the reprogrammed iPS cells consistent with the fact that they are maintained episomally, which is an important safety advantage. In addition, plasmid vectors can be manufactured and qualified for GMP use at a fraction of the cost of Sendai viral vectors. 

On the other hand, the mRNA reprogramming method offers the advantage of being completely free of genomic integration and is therefore highly suitable for clinical translation. However, the mRNA method was very inefficient at reprogramming primary patient fibroblasts consistent with the experience of several groups [21-23]. Therefore, further optimisation of this method is clearly required. Indeed our studies suggested that the inclusion of microRNA in the reprogramming cocktail did improve the reprogramming frequency of primary fibroblasts. Another disadvantage of the RNA based reprogramming method is that it is highly demanding, requiring daily transfections for 12 days, thus making it more difficult to develop a GMP compliant process. 

Although we found differences in reprogramming efficiencies between the plasmid and mRNA methodologies, we did not observe any qualitative differences in the iPS cell lines derived. The established iPS lines were compared using standard tests of pluripotency: expression of pluripotency markers using PCR and antibody staining, *in vitro* differentiation and *in vivo* differentiation potential through teratoma formation. In fact, all the iPS lines behaved similarly to the hES cell line, H1. We also compared our plasmid and mRNA derived iPS lines using the human pluripotent stem cell scorecard described by Meissner’s group [17]. This scorecard compares the gene expression pattern of key pluripotency and germ lineage markers relative to a reference standard that consists of 9 different human ES and iPS lines. Using cloud based software, we showed that our iPS lines derived using plasmids and mRNA were comparable to the reference standard and did not show any lineage bias. Indeed, the scorecard results confirmed our *in vitro* differentiation and teratoma assay results, suggesting that this focused array could potentially replace the more traditional tests of pluripotency. However, more partially and fully reprogrammed iPS lines will need to be fully analysed using the scorecard to demonstrate its efficacy.

Our findings show that there are no differences between iPS lines derived using plasmids and mRNA which supports a recent study comparing the hepatic differentiation potential of several iPS cell lines derived from different patients using episomal plasmids, Sendai virus or retrovirus, and showed that the major determinant of hepatic differentiation potential was the genetic background of patients and not the method of reprogramming [16]. While another study, showed that iPS lines generated from 5 different methods (3 factor vs 4 factor retroviral, lentiviral, episomal plasmid and mRNA) all acquired genetic mutations at the same frequency which was thought to be a consequence of extensive time in culture rather than the reprogramming method itself [24]. Taken together, these studies and our own suggest that the reprogramming method does not affect the quality of iPS cell lines produced.

Given the robustness of plasmid based reprogramming, we optimised this method further and showed that iPS cells can be derived using xeno-free ECMs and with defined media that is free of xeno-proteins without significant change in the reprogramming frequency. We achieved this by capitalising on the findings of two previous reports where one had demonstrated the generation of plasmid derived iPS lines in feeder free conditions using a complex cocktail of MEK-, GSK-3-, ROCK- and TGFβ- inhibitors while the other report showed that sodium butyrate improved the reprogramming efficiency of blood mononuclear cells (MNCs), though requiring the presence of feeders [25,26]. In contrast to other groups, we did not notice a significant decline in reprogramming efficiency with our feeder free protocol when compared to the feeder dependent method [27]. In addition, in our hands the plasmid method was very reproducible and capable of reprogramming primary fibroblasts from 5 different donors of varying ages (31-81 years old). All the patient iPS lines derived using plasmids possessed characteristics indicative of human pluripotent stem cells. This is consistent with what has been reported previously in the literature where a range of cell types including neonatal fibroblasts, dental pulp cells, adipose cells, CD34+ cells from cord blood and peripheral blood that have been successfully reprogrammed with this method [1,25,26]. While Karumbayaram et al. have reported the generation of patient iPS cells from skin biopsy using STEMCCA lentivirus and eventual differentiation into neurons under GMP compliant conditions, we are the first to show the generation of patient specific iPS cells using a non-viral method under feeder free conditions and with xeno-free reagents which can be easily transferred to a clean room environment with full GMP compliancy [10]. 

During the preparation of this manuscript, two ground breaking reports were published which could have major implications for iPS cell technology. The first report by Hou and colleagues showed that mouse iPS cells could be generated using a chemical cocktail of seven small compounds in 2i culture conditions without the addition of exogenous factors [28]. Small molecules are an attractive method of reprogramming, as their timing and delivery can be easily controlled by adjusting their concentrations in the culture medium. Although the caveat is that small molecules could elicit unknown off target effects, which may be no less detrimental than the transient expression of exogenous factors. In a different report, Jacob Hanna’s group achieved near 100% efficiency when generating mouse iPS cells from several different somatic cell types by eliminating the expression of the methyl-binding protein 3 (Mbd3), a key component of the nucleosome remodelling and deacetylation repressor complex (NuRD) [29]. However, such high efficiencies were only obtained in ‘secondary reprogrammable’ Mbd3 knock out somatic cells while siRNA knockdown of Mbd3 in wild type mouse embryonic fibroblasts only resulted in efficiencies of up to 18% [29]. While Mbd3 has been previously shown to be a roadblock to reprogramming, these findings are novel in that they support the notion of reprogramming being a deterministic process and not stochastic [30]. These studies clearly demonstrate that epigenetic modifiers are important for reprogramming and might be the key to finally establishing human iPS cells that are in the ground state of pluripotency. Further studies are needed to determine whether Mbd3 and other epigenetic modifiers serve to simply increase the efficiency of iPS cell generation or whether higher quality iPS cells can be generated with their use.

## Conclusions

This study is the first systematic evaluation of 3 reprogramming methodologies using the same set of cell lines and culture conditions which showed that episomal plasmids were the most suitable for developing a GMP compliant method for reprogramming primary patient fibroblasts. The robustness of our protocol was demonstrated by the high efficiency generation of 5 integration free and feeder free, patient specific iPS cell lines. We believe our protocol can be easily transferred to a clean room environment and hope that this will constitute a first step towards the derivation of GMP grade human iPS cell lines.

## Supporting Information

Figure S1 **Transduction and transfection efficiencies of fibroblasts.** A) GFP retroviral transduction of fibroblast cell lines. Scale bar on images is 200µm. Flow cytometric profiles show percentage of cells that are GFP positive. B) GFP transfection efficiency of fibroblast cell lines using the episomal plasmid. Scale bar on images is 200µm. Flow cytometric profiles show percentage of cells that are GFP positive. C) Overlay and individual Axiovision images of BJ fibroblasts transfected with GFP mRNA and counter stained with hoescht H33342.
(TIF)

Figure S2 **Q-PCR of embryoid bodies (EBs) and DNA fingerprinting.** A) Q-PCR showing the down regulation of pluripotency genes, Oct4 and Nanog in differentiated (diff) EBs relative to iPS lines. All results were normalised to the GAPDH housekeeping gene. B) Q-PCR showing the up regulation of lineage specific genes: Cdx2 (mesoderm), Mixl (mesendoderm) and Pax6 (ectoderm) in EBs relative to iPS lines. All results were normalised to the GAPDH housekeeping gene. C) DNA fingerprinting of iPS lines and starting parental fibroblasts, 3 loci are shown.
(TIF)
