# Supplementary figures and images for: A Systematic Evaluation of Integration Free Reprogramming Methods for Deriving Clinically Relevant Patient Specific Induced Pluripotent Stem (iPS) Cells
Source: PLoS One. 2013 Nov 26;8(11):e81622. doi: 10.1371/journal.pone.0081622 (PMC3841145; doi:10.1371/journal.pone.0081622)

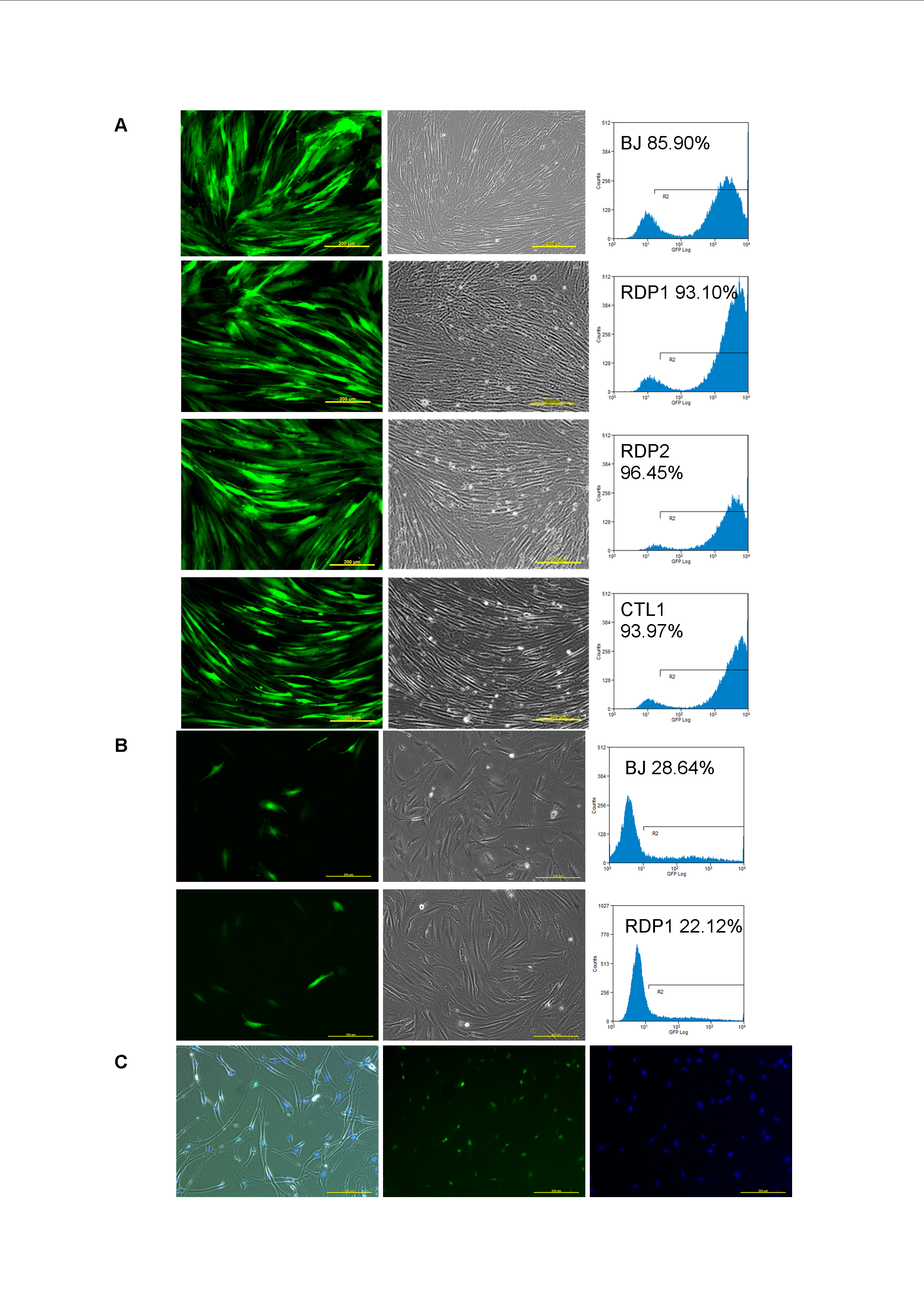

Supplement: Figure S1 — Transduction and transfection efficiencies of fibroblasts. A) GFP retroviral transduction of fibroblast cell lines. Scale bar on images is 200µm. Flow cytometric profiles show percentage of cells that are GFP positive. B) GFP transfection efficiency of fibroblast cell lines using the episomal plasmid. Scale bar on images is 200µm. Flow cytometric profiles show percentage of cells that are GFP positive. C) Overlay and individual Axiovision images of BJ fibroblasts transfected with GFP mRNA and counter stained with hoescht H33342. (TIF) [file pone.0081622.s001.tif]

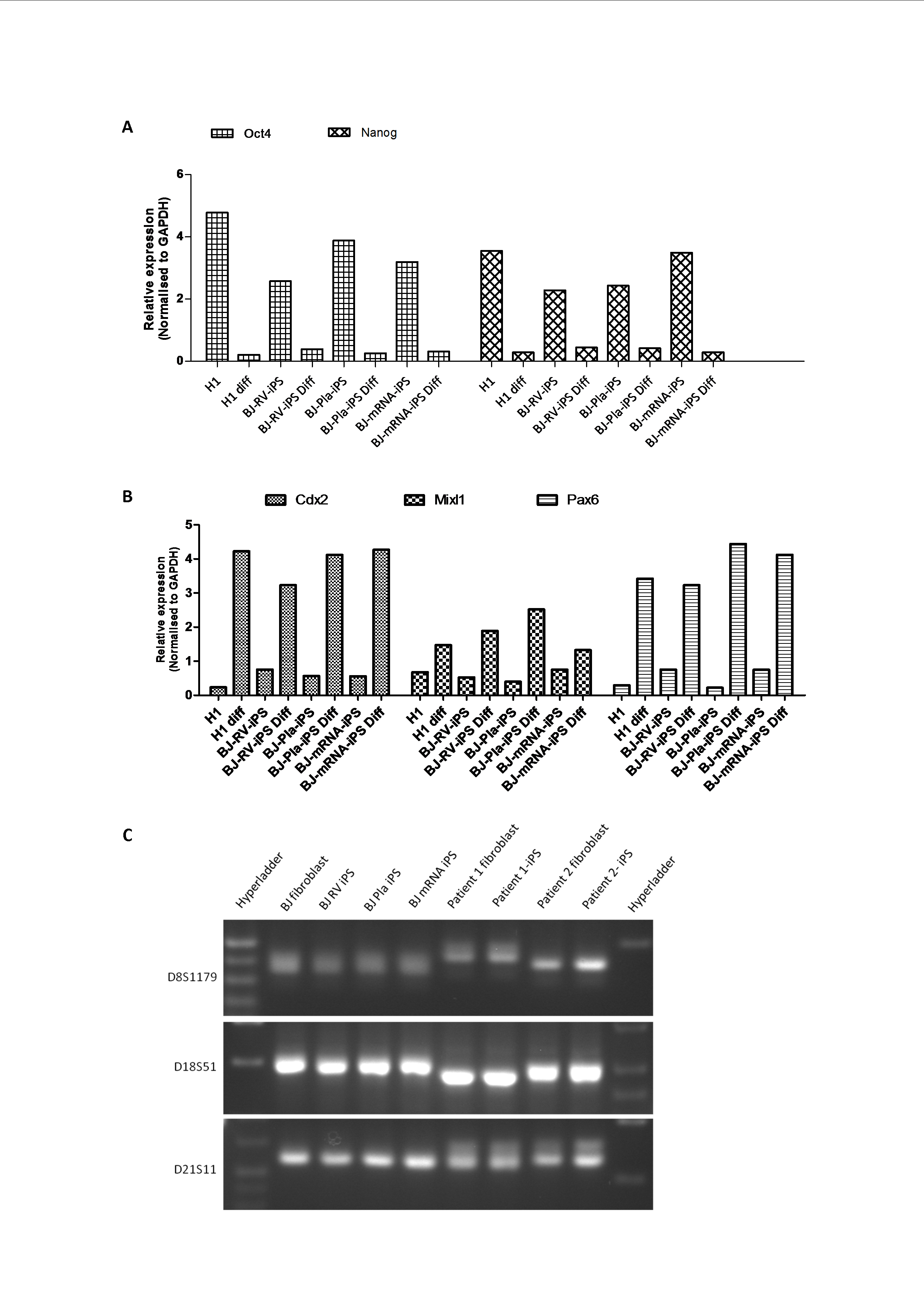

Supplement: Figure S2 — Q-PCR of embryoid bodies (EBs) and DNA fingerprinting. A) Q-PCR showing the down regulation of pluripotency genes, Oct4 and Nanog in differentiated (diff) EBs relative to iPS lines. All results were normalised to the GAPDH housekeeping gene. B) Q-PCR showing the up regulation of lineage specific genes: Cdx2 (mesoderm), Mixl (mesendoderm) and Pax6 (ectoderm) in EBs relative to iPS lines. All results were normalised to the GAPDH housekeeping gene. C) DNA fingerprinting of iPS lines and starting parental fibroblasts, 3 loci are shown. (TIF) [file pone.0081622.s002.tif]
